# Supplementary material for: Iron imaging in myocardial infarction reperfusion injury
Source: Nat Commun. 2020 Jun 29;11:3273. doi: 10.1038/s41467-020-16923-0 (PMC7324567; doi:10.1038/s41467-020-16923-0)
Supplement: Supplementary file 1 — Supplementary Information [file 41467_2020_16923_MOESM1_ESM.pdf]

Iron imaging in myocardial infarction reperfusion injury

Moon et al.

Supplementary Information

Supplementary Table 1. Cardiac function and infarct data by occlusion time at 1-week post-infarction

	45 min (n=3)	90 min (n=5)	180 min (n=5)	Per (n=4)	P *
Function (Cine MRI)					
LV mass (g)	79.9±7.6	84.8±10.3	95.3±29.9	86.9±17.4	0.87
EDV (mL)	105.4±12.5	107.0±22.3	104.5±17.2	114.4±20.9	0.64
ESV (mL)	57.8±4.0	58.8±16.8	53.2±10.5	60.9±19.0	0.73
EF (%)	45.0±2.7	45.7±4.6	47.6±17.3	47.4±10.7	0.85
CO (mL/min)	5.0±0.1	5.5±0.6	5.6±2.6	6.0±1.0	0.73
Infarct (LGE MRI) ‡					
Infarct size (g)	0.1±0.1	9.1±8.1	17.3±6.0	15.2±10.0	0.05
Transmurality (%)	5.1±7.1	54.3±12.1	72.6±8.6	79.4±15.1	0.02
MVO (g)	0.0±0.0	1.2±1.3	3.2±1.9	2.1±1.8	0.05
Magnetic susceptibility (QSM) §					
Iso $\Delta\chi$(SI)	0.007±0.024	-0.003±0.031	0.009±0.006	0.004±0.017	0.9
Hypo $\Delta\chi$(SI) #	NA	0.093±0.038	0.095±0.03	NA	0.96
Hyper $\Delta\chi$(SI)	0.03±0.049	0.021±0.015	0.01±0.009	0.038±0.013	0.58
P†	0.51	<0.001	0.004	0.02	

Relaxation time mapping (T2*)					
Iso *T2** [msec]	46.0±2.2	47.5±4.3	46.4±0.6	45.1±2.6	0.72
Hypo *T2** [msec] [€]	NA	14.0±2.9	14.1±2.8	NA	0.96
Hyper *T2** [msec]	54.3±17.4	55.2±13.8	68.7±3.63	53.3±12.7	0.36
P[†]	0.46	<0.001	<0.001	0.07	
Iron (ICP-OES) [‖]					
Remote myocardium [Fe]	0.038±0.006	0.0354±0.003	0.038±0.008	0.063±0.037	<0.001[**]
Infarcted myocardium [Fe]	0.075±0.041	0.108±0.068	0.184±0.066	0.119±0.145	<0.001
P[†]	NS	<0.001	<0.001	<0.001	

[*]Kruskal-Wallis test by occlusion time (45-, 90-, 180-min and permanent infarction) and by [†]location (infarct/remote or iso/hyper/hypo). Results are reported as mean±SD. Source data are provided as a Source Data file.

[‡]One animal in the 90-min coronary occlusion group did not undergo LGE MRI.

[§]Two animals in 180-min coronary occlusion group did not undergo explant MRI due to unavailability of the MRI scanner.

[‖]Two animals in the 90-min coronary occlusion group did not have ICP-OES analysis due to unavailability of the equipment.

[#]Hypointense regions were not observed in 45-min or permanent infarcts.

[**]Excluding the permanent infarction group the single-factor ANOVA results in P-value = 0.15.

LV = left ventricle, EDV = end diastolic volume, ESV = end systolic volume, EF = ejection fraction, CO = cardiac output, MVO = microvascular obstruction, Per = permanent ligatio

Supplementary Table 2. Cardiac function and infarct data by post-infarction time point

	baseline (n=5)	1 week (n=15)	8 week (n=8)	*P
Function (Cine MRI)				
LV mass (g)	82.9±18.7	85.4±14.2	128.7±15.7	<0.01
EDV (mL)	57.8±7.5	101.7±16.0	138.6±13.0	<0.01
ESV (mL)	25.5±9.1	62.4±13.2	97.0±14.3	<0.01
EF (%)	56.7±10.5	38.1±12.9	30.3±5.6	0.01
CO (L/min)	4.3±0.7	4.3±1.8	3.9±0.6	0.73
LVRI (L/min)	1.5±0.4	0.8±0.1	0.9±0.1	<0.01
Wall thickening (%)				
Remote	50.2±9.3	34.7±15.4	42.5±15.1	0.18
Infarct		9.9±13.9	15.4±17.3	0.64
†P		0.00	0.01	
ED wall thickness (mm)				
Remote	8.9±1.8	7.6±1.0	9.8±1.5	0.04
Infarct		7.5±1.1	6.6±0.9	0.01
†P		0.95	<0.01	
ES wall thickness (mm)				
Remote	13.2±3.1	10.1±1.0	13.6±1.7	<0.01
Infarct		7.8±1.6	7.5±1.2	0.04
†P		<0.01	<0.01	
Wall motion (mm)				
Remote	5.4±1.4	4.5±1.6	5.3±1.1	0.34
Infarct		2.3±1.3	2.5±1.0	0.72
†P		<0.01	<0.01	
Infarct (LGE MRI)				
Infarct size (g)		19.3±12.5	27.4±5.4	0.19
Transmurality (%)		61.6±12.1	52.9±3.1	0.11

MVO (g)		2.0±1.7	0.3±0.3	<0.01
Magnetic susceptibility (*Δχ*)				
myo (ppm)		-0.01±0.02	-0.01±0.04	0.13
[‡]inf (ppm)		0.06±0.05	-0.01±0.04	0.01
[†]P		0.01	1.00	
Iron (ICP-OES)[ǀ]				
Remote myocardium [Fe]		0.04±0.01	0.04±0.01	1
Infarcted myocardium [Fe]		0.15±0.08	0.06±0.04	<0.001
[†]P		<0.001	0.99	

[*]Indicates results of significance testing at p=0.05 level of significance using a Kruskal-Wallis test by post-infarction MRI time point and by [†]location (infarct/remote or iso/hyper/hypo).

[‡]Indicates region of elevated magnetic susceptibility inside the area of enhancing signal on late gadolinium enhanced MRI.

[ǀ]Seven animals in the 1-week timepoint group did not have ICP-OES analysis due to a subset of the animals being in a serial study and six animals in the 8-week timepoint group did not have ICP-OES due to unavailability of the equipment.

Results are reported as mean±SD. Source data are provided as a Source Data file.

Supplementary Table 3. Clinical and angiographic characteristics for all patients by presence of hemorrhagic infarction[a] (n=7)

	All Patients (n=7)	No myocardial hemorrhage (T2* core negative, n=3)	Myocardial hemorrhage (T2* core positive, n=4)
Clinical			
Age, y	61 [55,67]	61.7 [55.0, 69.0]	60.5 [55.5, 63.5]
Male sex, n (%)	6	2 (66)	4 (100)
BMI, kg/m^2	27.4 [25.3, 27.9]	28.3 [25.2, 30.1]	26.8 [22.8, 27.6]
History			
Hypertension, n (%)	5	2 (67)	3 (75)
Current smoker, n (%)	2	1 (33)	1 (25)
Hypercholesterolemia, n (%)	4	3 (100)	1 (25)
Diabetes mellitus[†], n (%)	2	1 (33)	1 (25)
Previous angina, n (%)	2	2 (66)	0 (0)
Previous myocardial infarction, n (%)	1	1 (33)	0 (0)
Previous PCI, n (%)	0	0 (0)	0 (0)
Presenting characteristics			
Heart rate, bpm	78.7 [70.0, 86.0]	84 [74, 96]	74.8 [70.0, 77.2]
Systolic bp, mmHg	129.7 [121.5, 136.5]	135.7 [126.0, 143.5]	125.2 [118.5, 134.8]

Diastolic bp, mmHg	79.1 [73.5, 81.5]	80.7 [71.0, 87.0]	78.0 [75.3, 80.8]
Time from symptom onset to reperfusion, min[b]	527.6 [186.5, 370.0]	303.7 [267.0, 369.0]	695.5 [164.8, 812.2]
Ventricular fibrillation[‡], n (%)	1	0 (0)	1 (25)
NYHA heart failure class			
0	5	2	3
I/II	1	1	0
III/IV	1	0	1
Number of diseased arteries[§], n (%)			
1	5	3 (100)	2 (50)
2	1	0 (0)	1 (25)
3	1	0 (0)	1 (25)
TIMI coronary flow grade before PCI, n (%)			
0/1	3	0	3
2/3	4	3	1
TIMI coronary flow grade after PCI, n (%)			
0/1	0	0	0

2	0	0	0
3	7	3	4
Medical Therapy			
ACEI or ARB	3	2 (67)	1 (25)
Beta blocker	3	1 (33)	2 (50)
1st Troponin -T	2.44 [0.06, 1.86]	0.30 [0.08, 0.41]	4.0 [0.04 5.6]
MRI			
LV mass, g	164.0 [140.5, 186.5]	146.7 [118.5, 169.5]	177.0 [158.5, 185.0]
EDV, mL	160.6 [144.5, 181.0]	135.3 [111.0, 168.0]	179.5 [167.8, 189.8]
EDVI, mL/m^2	79.3 [74.5, 91.5]	69.3 [58.0, 81.5]	86.8 [82.5, 91.8]
EF, %	51.7 [44.5, 55.0]	49.7 [48.0, 53.0]	53.3 [45.0, 59.8]
SV, mL	71.4 [57.5, 87.0]	66.0 [51.0, 87.5]	75.5 [71.0, 83.5]
Infarct size, g	39.9 [21.7, 57.7]	31.9 [14.0, 43.9]	45.9 [38.6, 52.8]
Transmurality, %	20.6 [3.4, 25.3]	48.8 [46.7, 51.6]	75.7 [65.9, 94.3]
MVO, g	6.0 [1.3, 9.5]	2.2 [0.3, 3.4]	8.9 [6.4, 12.0]
Magnetic susc. (rem. myocardium), ppm	-0.01 [-0.05, -0.2]	0.04 [0.02, 0.06]	-0.04 [-0.06,-0.03]
Magnetic susc. (hem. myocardium), ppm	0.16 [0.11, 0.23]	NA	0.16 [0.11 0.23]

[a]Continuous variables are reported as mean [1st, 3rd quartile].

[b]Time-to-reperfusion self-reported by patient in the emergency room.

[a]Hemorrhage was visually defined as a hypointense area >1 g tissue in T2*-weighted image corresponding to the region of myocardial infarction as assessed by late gadolinium enhanced MRI.

[†]Diabetes mellitus was defined as a history of diet-controlled or treated diabetes.

[‡]Successfully electrically converted ventricular fibrillation at presentation or during emergency PCI procedure.

[§]Multivessel coronary artery disease was defined according to the number of stenoses of at least 50% of the reference vessel diameter, by visual assessment and whether or not there was left main involvement.

LV = left ventricle, EDV = end diastolic volume, EDVI = end diastolic volume index, EF = ejection fraction, SV = stroke volume, MVO = microvascular obstruction.

Supplementary Table 4. Cardiac function and infarct data for all patients (n=7)

Subject ID	LV mass, g	EDV, mL	EDVI, mL/m^2	EF, %	SV, mL	Infarct size, g (%)	transmurality (%)	MVO, g (%)	Hemorrhage (yes/no)	Remote myocardium magnetic susceptibility (ppm)	Hemorrhagic myocardium magnetic susceptibility (ppm)
1	145	137	78	57	78	23.5 (14.6)	36.0	2.12 (1.31)	Yes	0.006±0.2	0.14±0.23
2	170	178	84.0	46	80	43.6 (32.2)	75.9	7.8 (5.8)	Yes	-0.08±0.4	0.22±0.6
3	136	184	92	53	96	19.9 (12.9)	43.4	0.5 (0.3)	No	0.01±0.9	NA
4	203	152	71	43	65	67.9 (42.6)	53.2	6.2 (3.9)	No	0.02±0.5	NA
5	101	70	45	53	37	8.0 (10.1)	50.0	0.0 (0.0)	No	0.09±0.4	NA
6	230	225	94	42	94	68.8 (38.9)	93.0	11.2 (6.3)	Yes	-0.05±0.3	0.03±0.2
7	163	178	91	68	50	47.5 (44.5)	98.0	14.5 (13.0)	Yes	-0.04±0.3	0.24±0.4

LV = left ventricle, EDV = end diastolic volume, EDVI = end diastolic volume index, EF = ejection fraction, CO = cardiac output, MVO = microvascular obstruction. Hemorrhage was visually defined as a hypointense area >1 g tissue in T2*-weighted image corresponding to the region of myocardial infarction as assessed by late gadolinium enhanced MRI. Magnetic susceptibility results are reported as mean±SD.

Supplementary figures

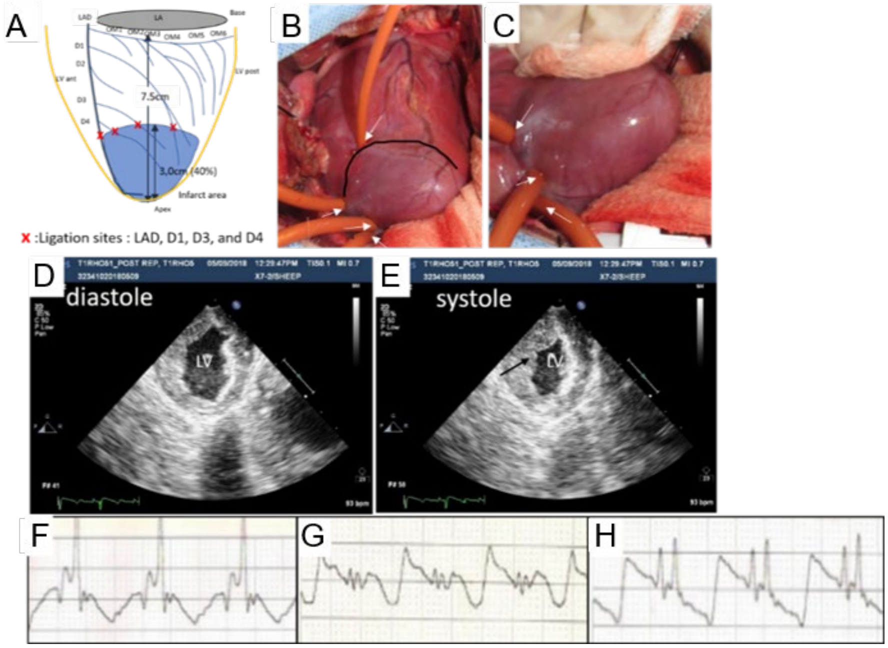

Supplementary Figure 1. Generation of the animal model.

Myocardial infarct generation in one animal with 90 minutes of coronary artery occlusion followed by reperfusion. (**A)** Schematic of the left ventricle coronary artery anatomy (red cross indicates suture sites for coronary occlusion). (**B)** Myocardial ischemia observed by direct inspection of visual discoloration of heart muscle to the affected area. White arrows indicate suture points for coronary occlusion in apical view. Solid black line indicates the infarct borderzone. (**C)** Medial view (**D)** echocardiogram shows hypokinetic wall motion (black arrows) at diastole and (**E**) systole. (**F)** electrocardiogram (ECG) observed at baseline (**G)** ST-elevation myocardial infarction at 90-min of coronary occlusion with limited definition of QRS complex (**H)** partial restoration of QRS complex 30 minutes after reperfusion. LAD = left anterior descending coronary artery. D1-4 = first through fourth diagonal branches of LAD. OM1-OM6 = obtuse marginal branches of left circumflex artery. LA = left atrium. LV = left ventricle.

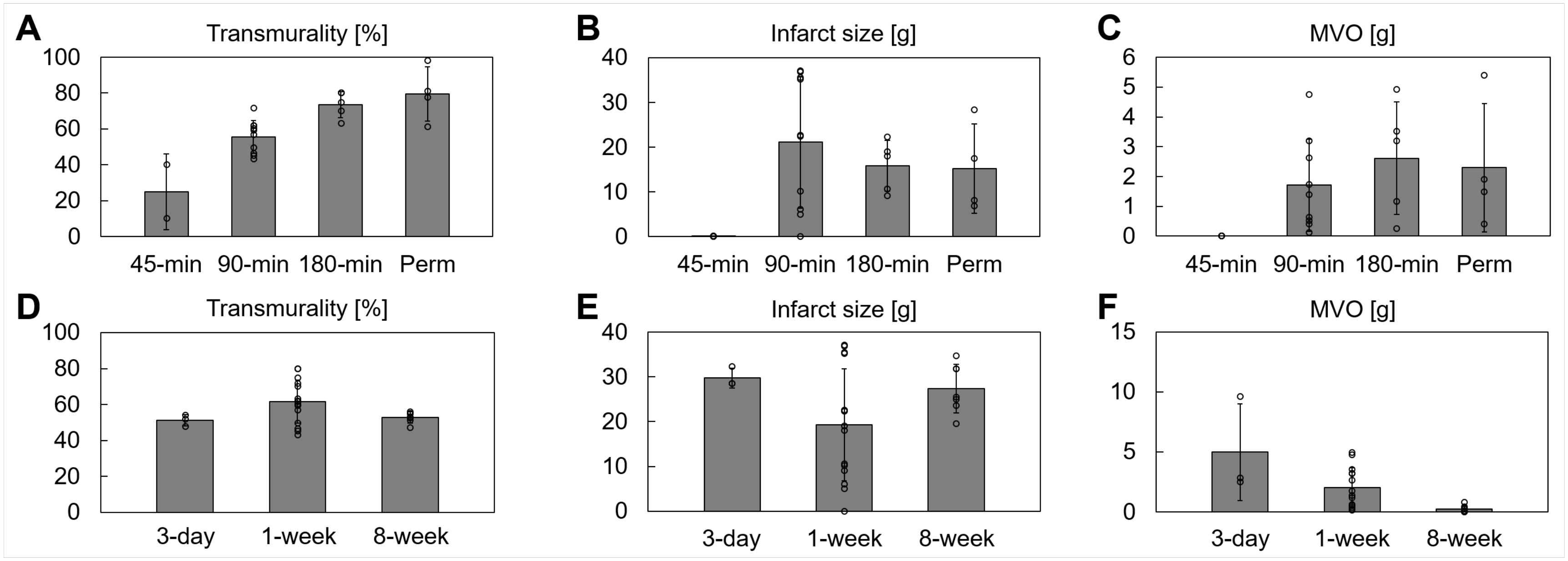

Supplementary Figure 2. Infarct transmurality, size and microvascular obstruction.

Infarct data is displayed by (**A-C**) time-to-reperfusion (45-, 90-, 180-min and permanent occlusion) at 1-week post-infarction and by (**D-F**) post-infarction timepoint (3-day, 1-week, 8-week) after a 90-, 180-min coronary occlusion. Infarct properties from animals as determined by late gadolinium enhanced MRI (n=31), (**A,D**) infarct transmurality, (**B,E**) infarct size, (**C,F**) microvascular obstruction (MVO). Two animals (of 33 total) did not undergo LGE MRI. Results are reported as mean±SD. Source data are provided as a Source Data file.

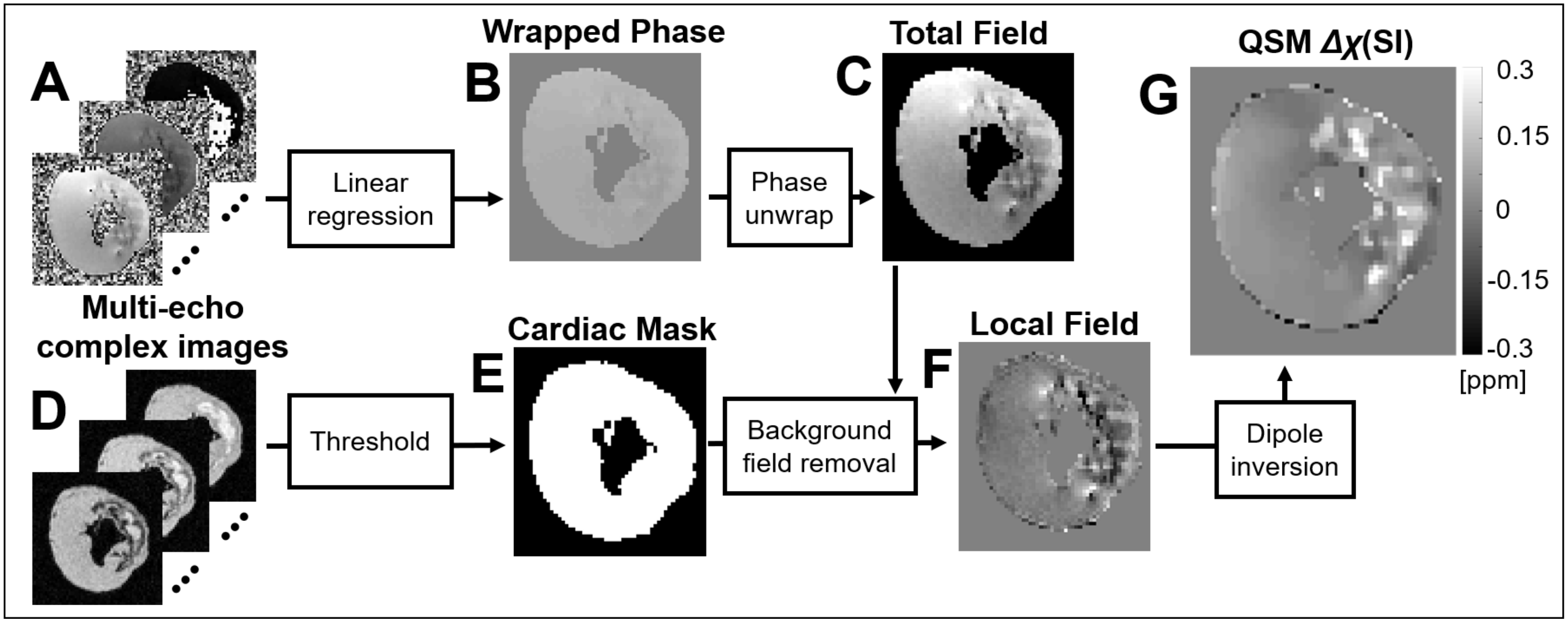

Supplementary Figure 3. Generation of quantitative susceptibility maps from gradient echo MRI data.

(**A**) gradient echo phase images at several echo times. (**B**) phase images are unwrapped to remove phase aliasing. (**C**) total field map was obtained by voxel-based linear regression. (**D**) magnitude images are thresholded to remove image noise to generate (**E**) a cardiac binary mask. (**F**) local fields were estimated using the projection onto dipole field (PDF) background field removal algorithm and (**G**) quantitative susceptibility maps were generated by morphology enabled dipole inversion (MEDI) as described in online Methods.

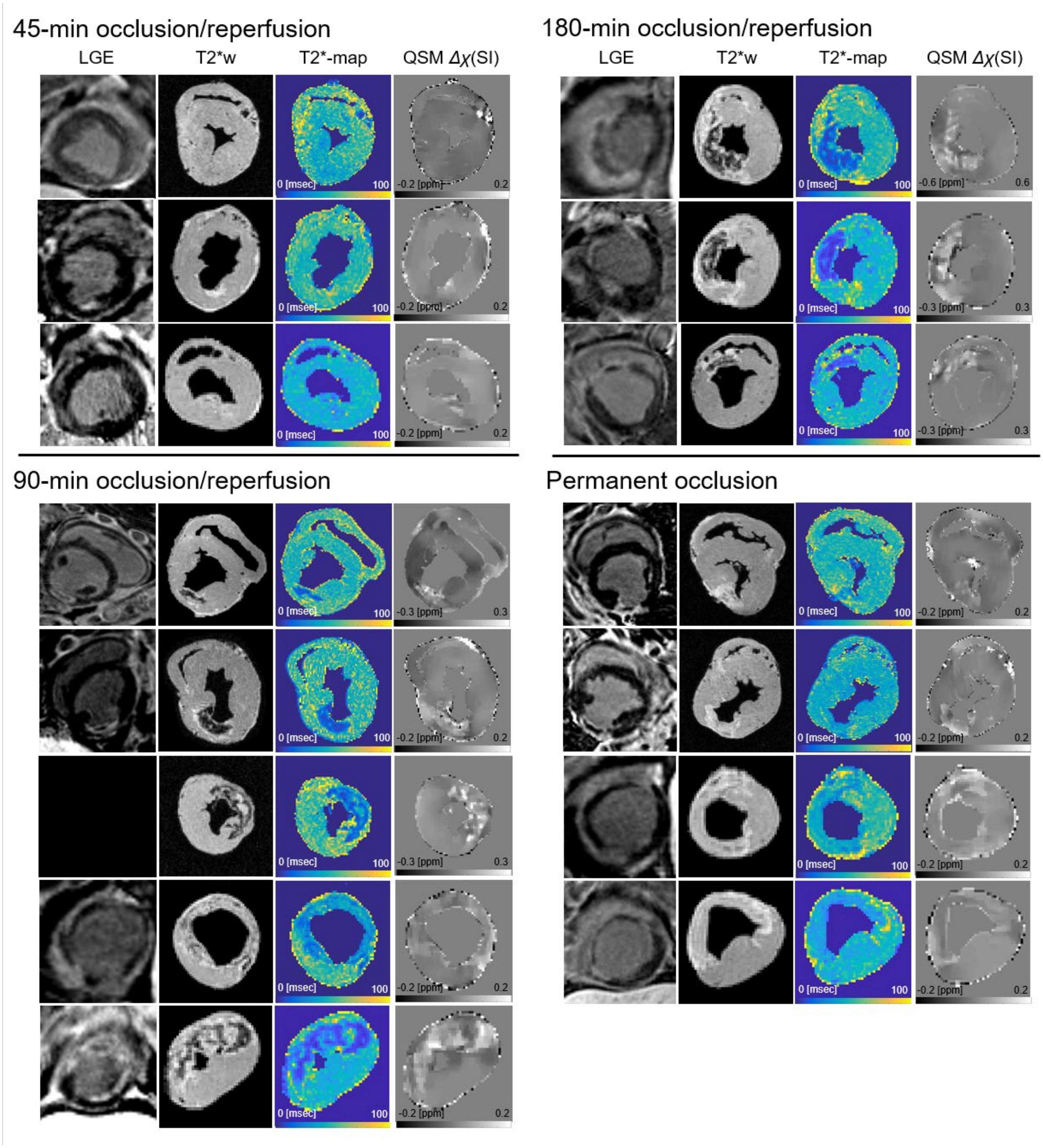

Supplementary Figure 4. Ex vivo MRI data from all animal models.

Short axis cardiac images are displayed by time-to-reperfusion (45-, 90-, 180-min, permanent occlusion) and post-infarction time point (3-day, 1-week, 8-week). Late gadolinium enhanced (LGE) MRI (column 1), T2*-weighted images (TE=16.1 msec) (column 2), T2*-maps (column 3), quantitative magnetic susceptibility maps (column 4).

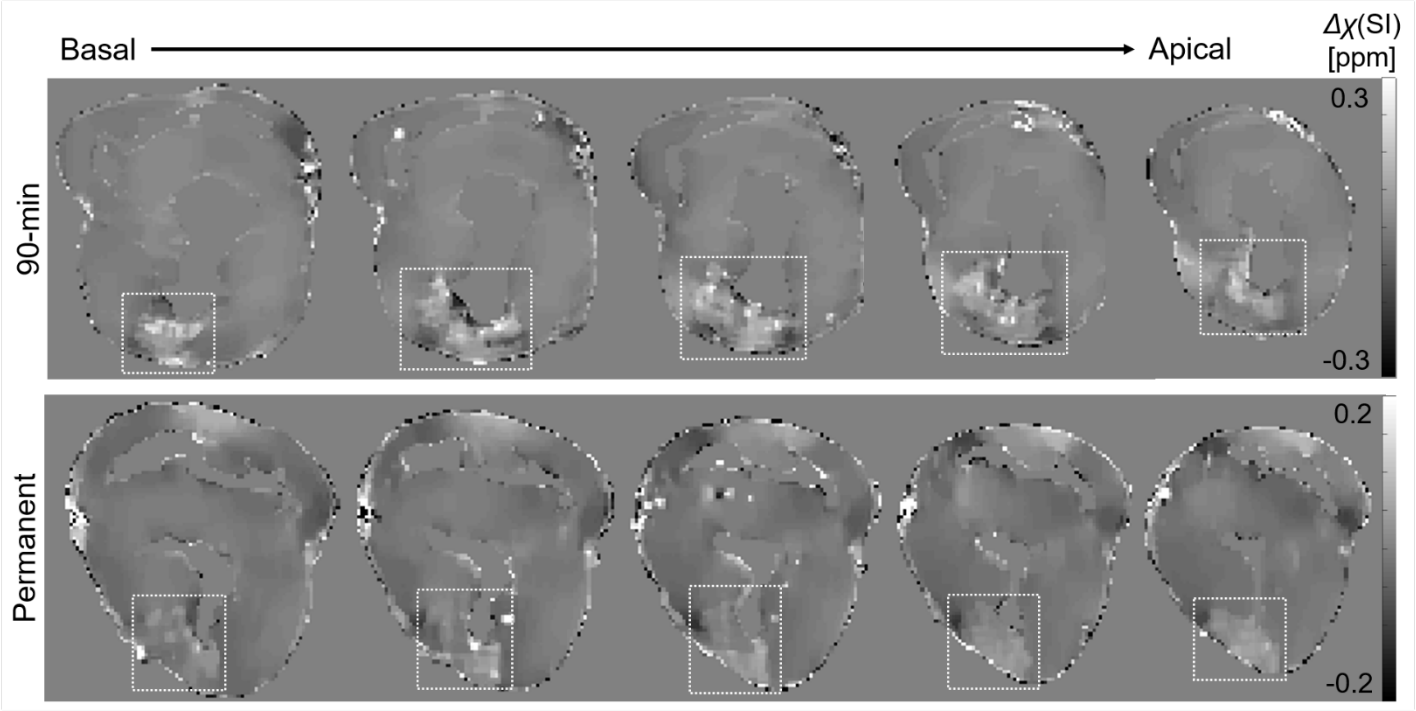

Supplementary Figure 5. Basal to apical consecutive ex vivo quantitative susceptibility maps.

Representative quantitative susceptibility maps from 90-min occlusion and reperfusion (first row) and permanent occlusion (second row) animal models at 1-week post-infarction.

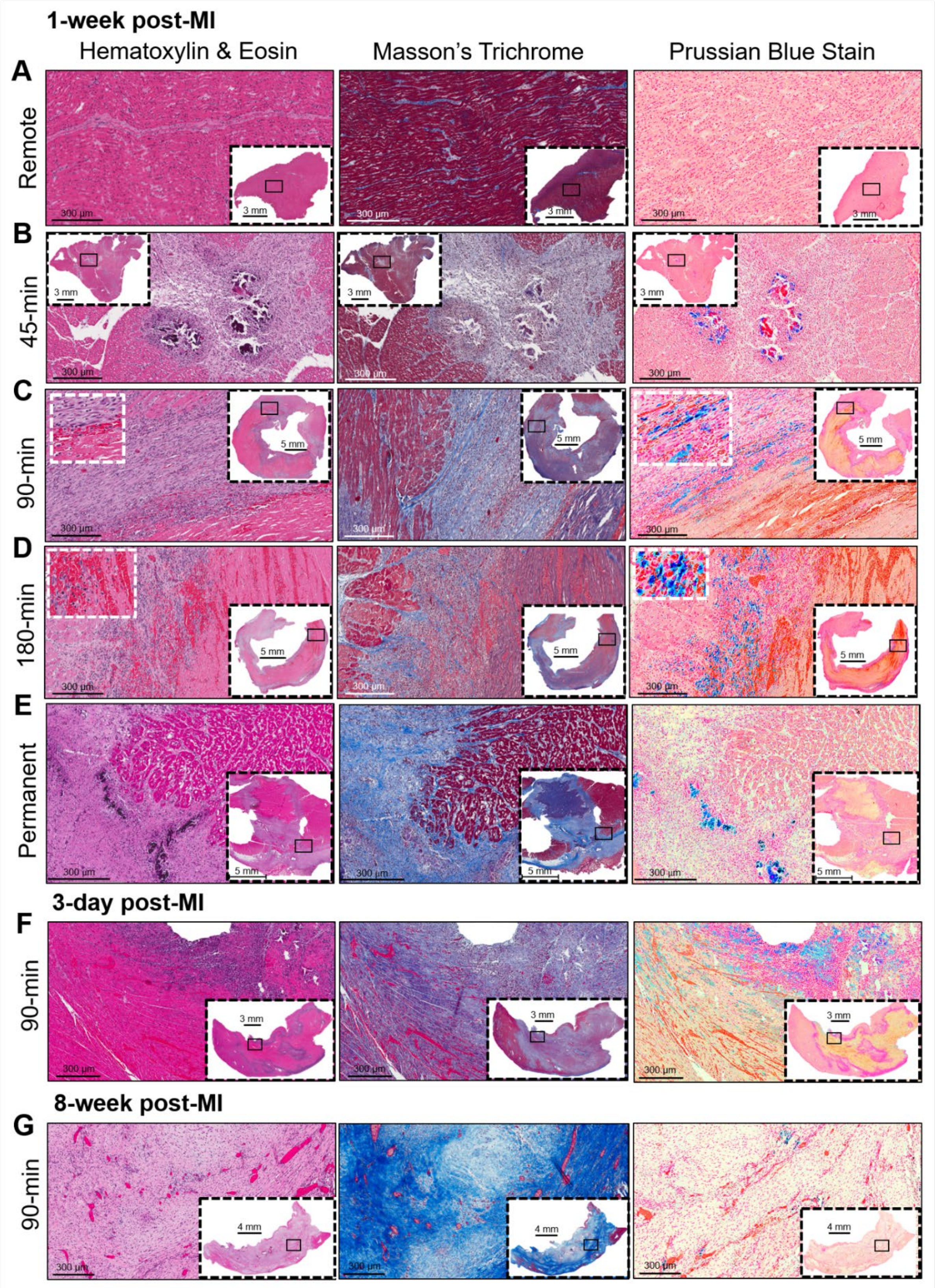

Supplementary Figure 6. Microscopy images from each animal model group.

Representative histology from each animal model group is displayed by time-to-reperfusion (45-, 90-, 180-min, permanent occlusion) and post-infarction time point (3-day, 1-week, 8-week) (n=7). Hematoxylin and eosin stain (column 1), Masson's trichrome stain (column 2), Prussian blue stain (column 3).

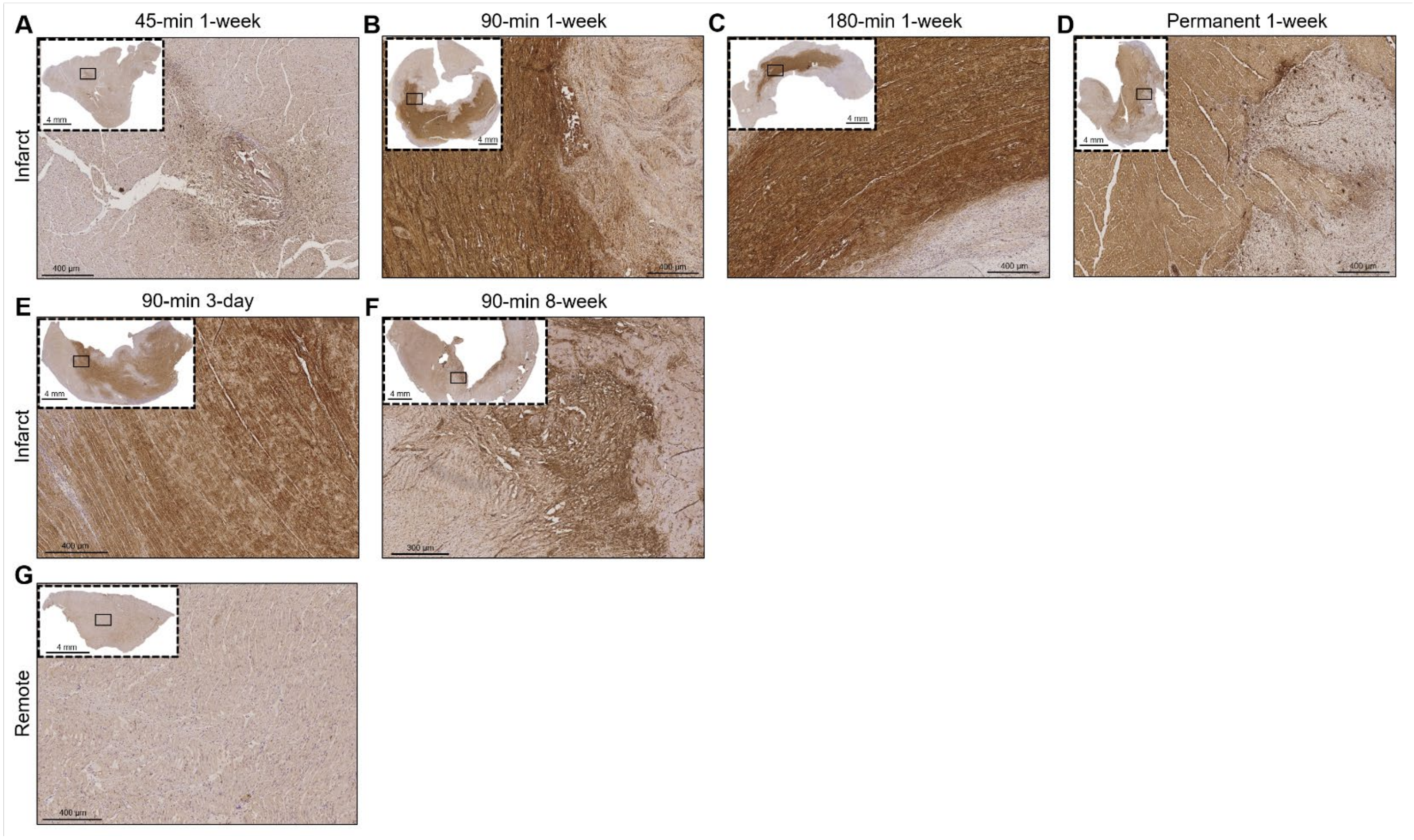

Supplementary Figure 7. Hemoglobin beta immunohistochemistry from each animal model group. Representative Hemoglobin beta (HBB) immunohistochemistry (IHC) HBB IHC from each animal model group is displayed by time-to-reperfusion (45-, 90-, 180-min, permanent occlusion) and post-infarction time point (3-day, 1-week, 8-week) in select animals (n=7). (**A**) 45-min 1-week infarct has darker staining in the infarct area without obvious concentration areas of positive HBB staining. (**B**) 90-min 1-week and (**C**) 180-min 1-week infarcts have concentration areas of positive HBB staining. The darker staining may reflect uptake and extracellular hemoglobin from past hemorrhage. Additionally, there are areas that show granular accumulation of hemoglobin corresponding to red blood cells. (**D**) Similar dark staining in the permanent infarct, with less concentrated areas of positive HBB staining. (**E**) 90-min 3-day infarct has positive HBB staining with granular accumulation of hemoglobin. The area of positive HBB declined by 8-week post-infarction as shown in (**F**) 90-min 8-week infarct, indicating a clearance of hemoglobin during wound healing. (**G**) Remote myocardium does not have positive HBB staining.

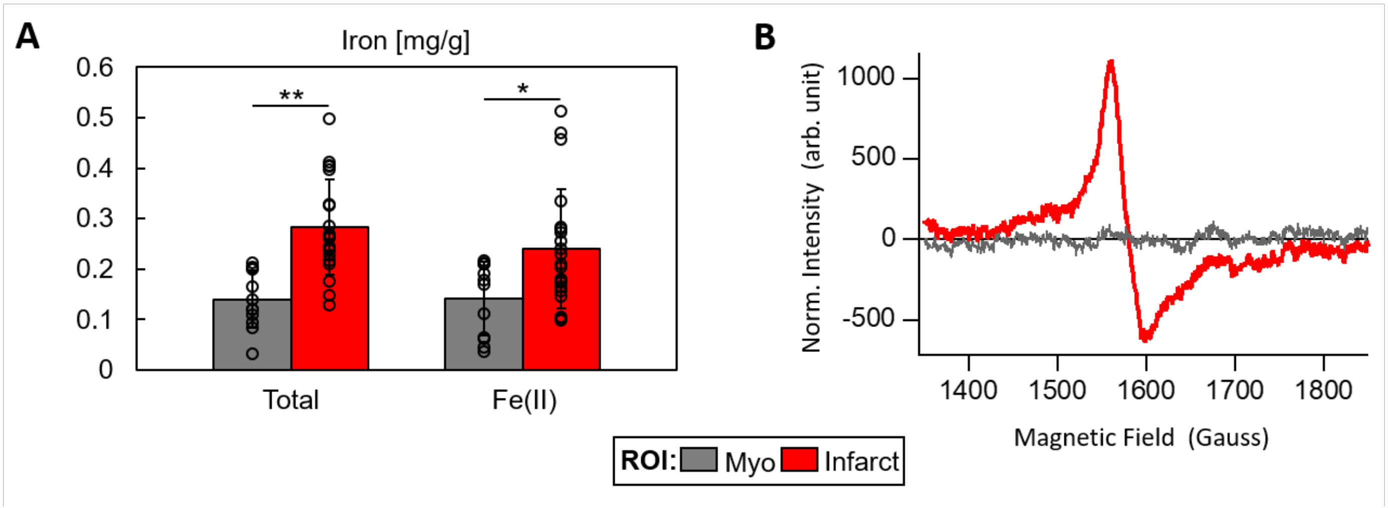

Supplementary Figure 8. Ex vivo Iron Measurements.

(**A**), Colorimetric measurement of total iron content revealed the same trend as the ICP-OES measurements (myo, n=13, infarct, n=22), where there was a significant increase in iron content within the infarct region ($P<0.0001$). Colorimetric measurement of Fe^{2+} iron showed the same trend as total iron ($P=0.006$) and suggested most of the iron in the tissue is Fe^{2+} iron. Tissue regions were compared using a two-tail student's two-sample t-test for both the total and Fe^{2+} iron measurements. Results are reported as mean±SD significance is indicated by *$P<0.05$ and **$P<0.001$ by t-test infarct vs. corresponding remote area. (**B**), Representative spectra of 180-min group infarct and remote area (myo) tissue specimens by electron paramagnetic resonance reflecting the tissue content of labile iron in Figure 2 A. Source data for A are provided as a Source Data file.

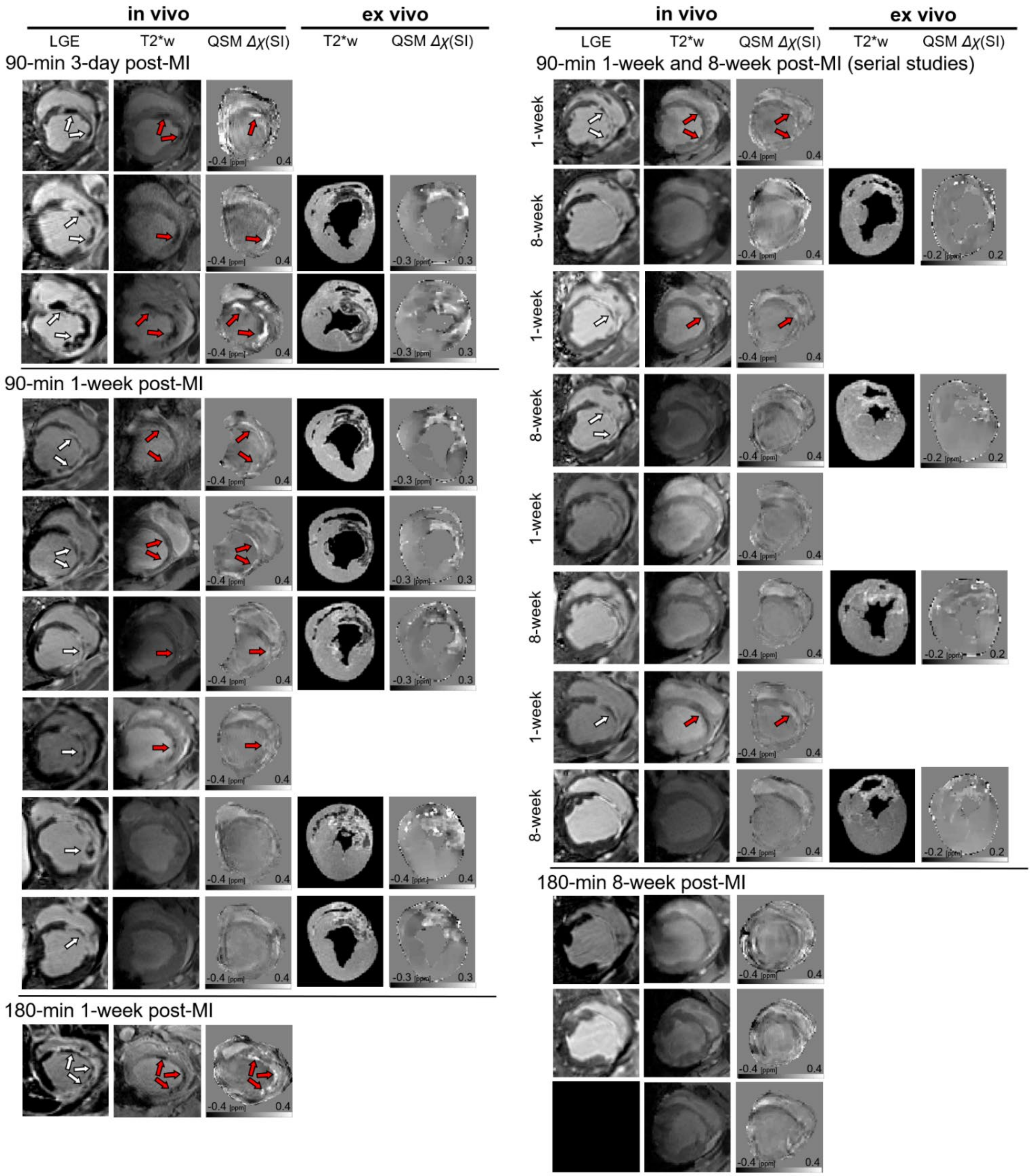

Supplementary Figure 9. In vivo MRI data from 90-, 180-min reperfused animal models.

Short axis cardiac images are displayed by time-to-reperfusion (90-, 180-min occlusion) and post-infarction time point (3-day, 1-week, 8-week) and a subset of corresponding ex vivo MRI. In vivo: late gadolinium enhanced (LGE) MRI (column 1), T2*-weighted images (column 2, TE=10 msec), quantitative magnetic susceptibility maps (column 3), ex vivo: T2*-weighted images (column 4, TE=16.1 msec), quantitative magnetic susceptibility maps (column 5). Each row shows the MRI from a single animal.

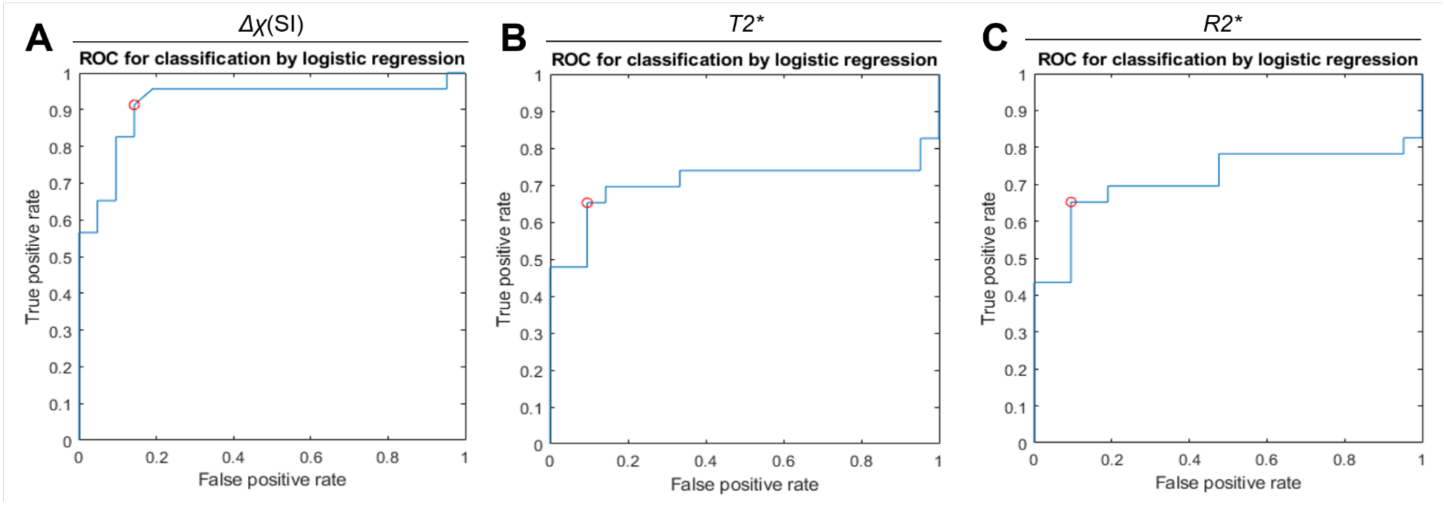

Supplementary Figure 10. Classification of infarct versus remote myocardium.

The predictor variables were (**A**) tissue magnetic susceptibility (*Δχ*), (**B**) transverse relaxation time (*T2**) and (**C**) transverse relaxation rate (*R2**) measurements that had a binary response variable as infarct (n=22) equal to 1 and remote myocardium (n=22) equal to 0 and were fit to a logistic regression model. *Δχ* provided the receiver operating characteristic (ROC) curve with an area under the curve (AUC) = 0.92, Youden's index = 0.77 and at the optimal operating point (red circle) sensitivity = 0.91 and specificity = 0.86. *T2** and *R2** ROC analysis provided an AUC= 0.71, Youden's index = 0.56 and at the optimal operating point sensitivity = 0.65 and specificity = 0.90. *Δχ* showed superior balance between sensitivity and specificity at classifying infarct versus remote viable myocardium compared to *T2** and *R2**. Source data are provided as a Source Data file.

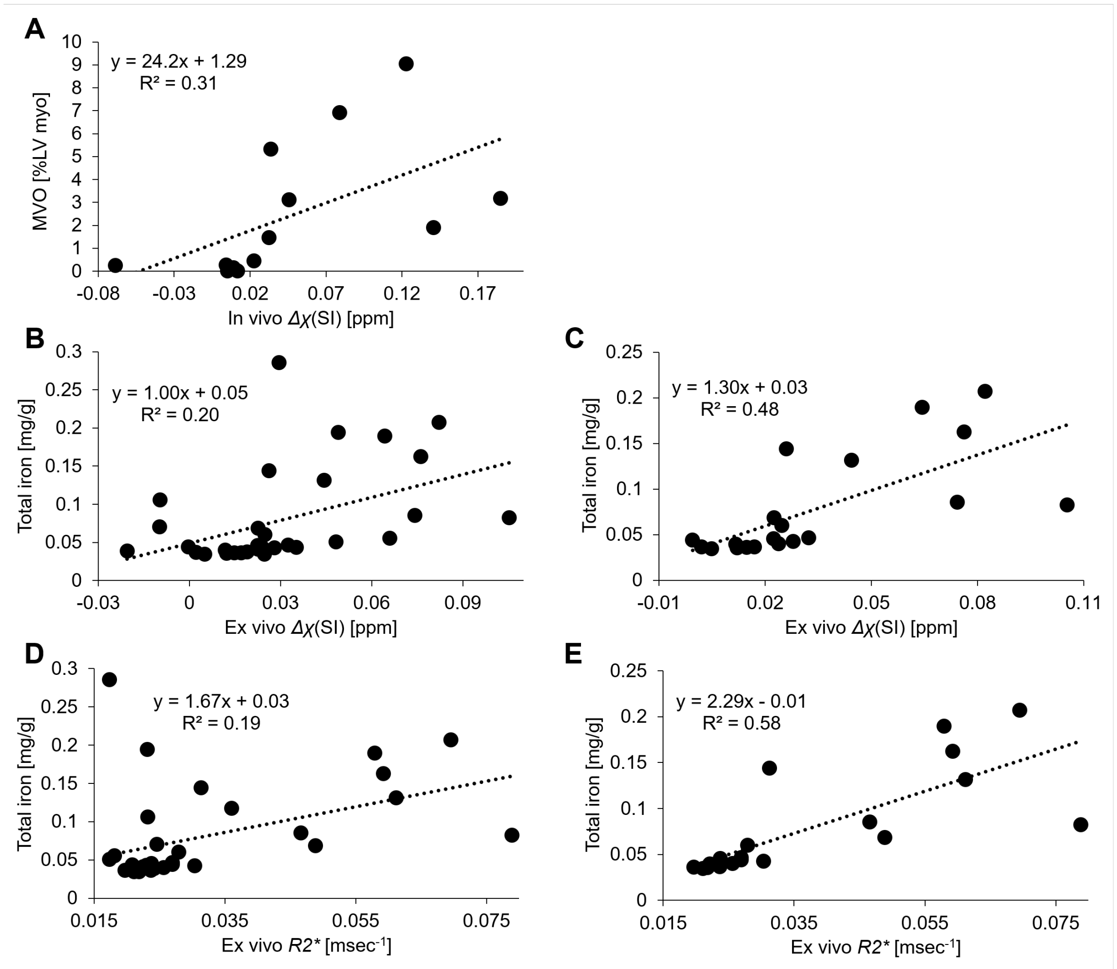

Supplementary Figure 11. Magnetic susceptibility, microvascular obstruction and iron. (**A**) microvascular obstruction (MVO), percentage of LGE infarct hypointense region with respect to LV volume, versus in vivo infarct magnetic susceptibility (slope=24.2% LV myo increase per 1 ppm increase in tissue magnetic susceptibility, R^2=0.31, P=0.048). (**B**) Infarct (45-, 90-, 180-min, permanent) and remote myocardium total iron concentration versus corresponding ex vivo tissue magnetic susceptibility (slope = 1.00 mg/g of total iron per 1 ppm increase in tissue magnetic susceptibility, R^2=0.20, P=0.009). (**C**) Infarct (90-, 180-min) and remote myocardium total iron concentration versus corresponding ex vivo tissue magnetic susceptibility (slope = 1.30 mg/g of total iron per 1 ppm increase in tissue magnetic susceptibility, R^2=0.48, P=0.0007). (**D**) Infarct (45-, 90-, 180-min, permanent) and remote myocardium total iron concentration versus corresponding ex vivo transverse relaxation rate (*R2**) (slope = 1.67 mg/g of total iron per 1 $msec^{-1}$ increase in *R2**, R^2=0.19, P=0.011). (**E**) Infarct (90-, 180-min) and remote myocardium total iron concentration vs corresponding ex vivo

tissue *R2** (slope = 2.29 mg/g of total iron per 1 $msec^{-1}$ increase in *R2**, R^2=0.58, P<0.0001). Linear regressions were performed to determine if there was a significant correlation based on the linear regression coefficient. All post-infarction time points (3-day, 1-week, and 8-week) were included in the data. Each point in the regressions represent the measurements from a single animal and total iron concentration was measured by ICP-OES. Source data are provided as a Source Data file.

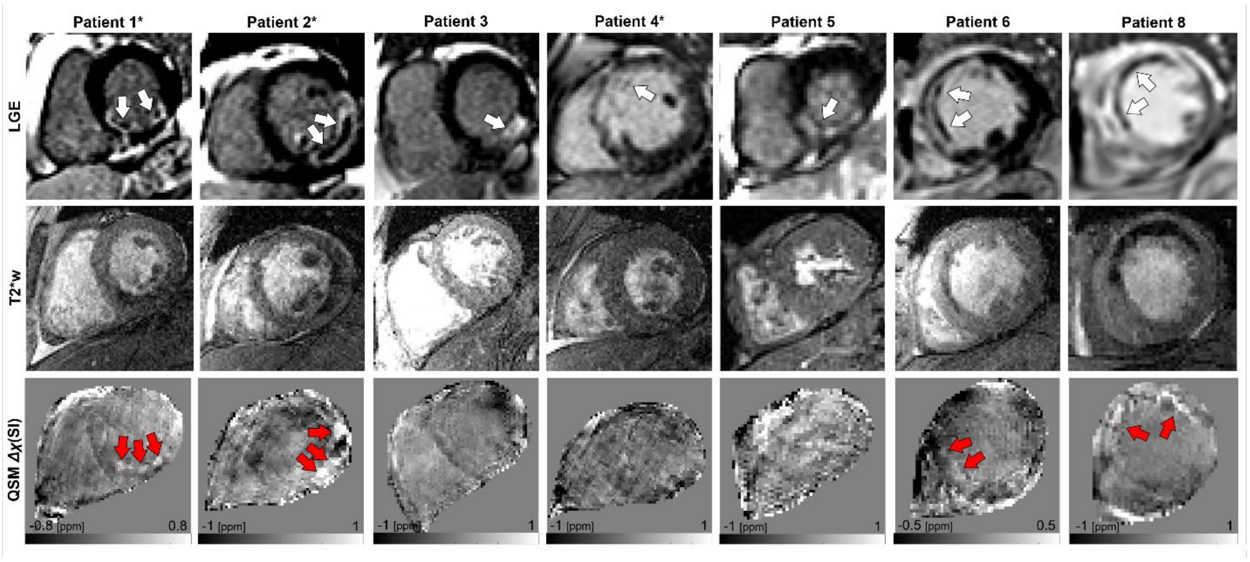

Supplementary Figure 12. Magnetic resonance imaging from all STEMI patients (n=7). Late gadolinium enhanced MRI (first row), T2*-weighted MRI (second row), quantitative susceptibility maps (QSM) (third row). * Patients are represented in Figure 6.

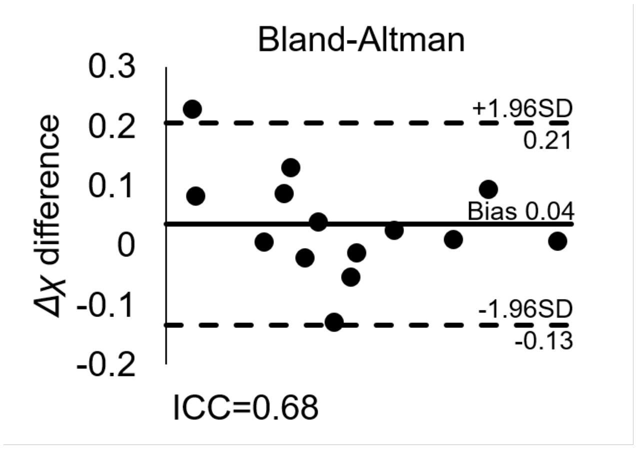

Supplementary Figure 13. Reproducibility of magnetic susceptibility in STEMI patients. Bland-Altman and intra-class correlation coefficient show no significant bias and good correlation between two raters. Remote and infarct myocardium magnetic susceptibility was measured in seven STEMI patients. Source data are provided as a Source Data file.
