# Supplementary material for: Iron imaging in myocardial infarction reperfusion injury
Source: Nat Commun. 2020 Jun 29;11:3273. doi: 10.1038/s41467-020-16923-0 (PMC7324567; doi:10.1038/s41467-020-16923-0)
Supplement: Supplementary file 3 — Reporting Summary [file 41467_2020_16923_MOESM3_ESM.pdf]

nature research

Corresponding author(s): Walter R. Witschey

Last updated by author(s): Apr 2, 2020

Reporting Summary

Nature Research wishes to improve the reproducibility of the work that we publish. This form provides structure for consistency and transparency in reporting. For further information on Nature Research policies, see Authors & Referees and the Editorial Policy Checklist .

Statistics

For all statistical analyses, confirm that the following items are present in the figure legend, table legend, main text, or Methods section.

n/a	Confirmed	
☐	☒	The exact sample size (n) for each experimental group/condition, given as a discrete number and unit of measurement
☐	☒	A statement on whether measurements were taken from distinct samples or whether the same sample was measured repeatedly
☐	☒	The statistical test(s) used AND whether they are one- or two-sided<br>*Only common tests should be described solely by name; describe more complex techniques in the Methods section.*
☐	☒	A description of all covariates tested
☐	☒	A description of any assumptions or corrections, such as tests of normality and adjustment for multiple comparisons
☐	☒	A full description of the statistical parameters including central tendency (e.g. means) or other basic estimates (e.g. regression coefficient) AND variation (e.g. standard deviation) or associated estimates of uncertainty (e.g. confidence intervals)
☐	☒	For null hypothesis testing, the test statistic (e.g. F, t, r) with confidence intervals, effect sizes, degrees of freedom and P value noted<br>*Give P values as exact values whenever suitable.*
☒	☐	For Bayesian analysis, information on the choice of priors and Markov chain Monte Carlo settings
☐	☒	For hierarchical and complex designs, identification of the appropriate level for tests and full reporting of outcomes
☒	☐	Estimates of effect sizes (e.g. Cohen's d, Pearson's r), indicating how they were calculated

Our web collection on statistics for biologists contains articles on many of the points above.

Software and code

Policy information about availability of computer code

Data collection	Animal Cardiovascular Magnetic Resonance Protocol: MRI studies were performed on a 3 T whole-body system (Trio; VB17 scanner; Siemens Healthcare, Erlangen, Germany) with 40 mT/m gradients and 18 channel RF receiver arrays. Intraventricular pressure was interfaced to physiological monitoring software and filtered to facilitate dual respiratory and cardiac gating (LabView, National Instruments, Inc., Austin Texas, versio 2018). All 2D images were acquired in the short-axis during apnea and 3D with dual cardiac and respiratory gating. Apnea was achieved by temporarily disabling the animal ventilator. Human Cardiovascular Magnetic Resonance Protocol: MRI studies were performed on a 1.5 T whole-body system (Aera; VE11C scanner; Siemens Healthcare, Erlangen, Germany) with 40 mT/m gradients and 48 channel RF receiver arrays. Electrocardiograms were obtained during MRI and used for gating using a wireless ECG monitor. All 2D images were acquired in the short-axis during breath-holding and 3D with dual cardiac and respiratory gating.
Data analysis	Source Code - Cornell MRI Research lab, Cornell University, Quantitative Susceptibility Mapping toolbox (http://pre.weill.cornell.edu/mri/pages/qsm.html). Source code - Penn Image Computing and Science Laboratory, University of Pennsylvania, ITK-SNAP (http://www.itksnap.org/pmwiki/pmwiki.php?n=Downloads.SNAP3). Source Code - Medis medical imaging systems, Leiden, The Netherlands, Qmass 7.5 (https://www.medis.nl/products/qmass). Custom Code - Advanced Cardiovascular Imaging Lab, University of Pennsylvania, T2* mapping (git clone https://moonbri@bitbucket.org/moonbri/penn_corlab_public_repository.git).

For manuscripts utilizing custom algorithms or software that are central to the research but not yet described in published literature, software must be made available to editors/reviewers. We strongly encourage code deposition in a community repository (e.g. GitHub). See the Nature Research guidelines for submitting code & software for further information.

Data

Policy information about availability of data

All manuscripts must include a data availability statement. This statement should provide the following information, where applicable:

- Accession codes, unique identifiers, or web links for publicly available datasets
- A list of figures that have associated raw data
- A description of any restrictions on data availability

All data is available upon reasonable request to the authors and approved through the University of Pennsylvania. The source data underlying Figures 1B-C, 2A-E, 3B-C, 5B-C, Supplementary Figures 2, 8A, 10, 11A-E, 13 and Supplementary Tables 1, 2 are provided as a source data file. There may be restrictions on private health data. Source data from Supplementary Tables 3 and 4 were removed to protect patient confidentiality. MRI performed in the context of this research study did not influence the treatment of the patients enrolled in the study.

Field-specific reporting

Please select the one below that is the best fit for your research. If you are not sure, read the appropriate sections before making your selection.

[x] Life sciences [ ] Behavioural & social sciences [ ] Ecological, evolutionary & environmental sciences

For a reference copy of the document with all sections, see nature.com/documents/nr-reporting-summary-flat.pdf

Life sciences study design

All studies must disclose on these points even when the disclosure is negative.

Sample size

The animal sample size from our study demonstrated a significant difference in susceptibility and iron concentration at different reperfusion times and post-infarction timepoints, similarly to the myocardial infarction reperfusion study performed by Llaneras et al, Markovitz et al, Moainie, et al. Therefore, we did not increase the swine (N=38) sample size: reperfusion at 45 (n=3), 90 (n=10), 180 (n=5) minutes after infarct onset and permanent occlusion (n=4) at 1-week post-infarction. Three additional groups were studied at 3-day (n=3) and 8-week (n=8) after a 90- or 180-min occlusion followed by reperfusion and a separate set of controls without myocardial infarction (n=5). The clinical study was performed to test the feasibility of cardiac quantitative susceptibility mapping in a cohort of seven STEMI patients. Therefore, sample size power analysis was not performed prior to the clinical study.

Llaneras, M.R., et al., Large animal model of ischemic mitral regurgitation. Ann Thorac Surg, 1994. 57(2): p. 432-9.
Markovitz, L.J., et al., Large animal model of left ventricular aneurysm. Ann Thorac Surg, 1989. 48(6): p. 838-45.
Moainie, S.L., et al., An ovine model of postinfarction dilated cardiomyopathy. Ann Thorac Surg, 2002. 74(3): p. 753-60.

Data exclusions

There were 38 animals, one animal in the 90 min reperfusion infarct group did not undergo LGE MRI, two animals in 180 min reperfusion infarct group did not undergo explant MRI due to unavailability of the MRI scanner, two animals in the 90 min reperfusion infarct group did not have ICP-OES analysis due to unavailability of the equipment. Seven animals in the 1-week timepoint group did not have ICP-OES analysis due to a subset of the animals being in a serial study and six animals in the 8-week timepoint group did not have ICP-OES due to unavailability of the equipment. The exclusion criteria were not pre-established, exclusion of animal data was due to unforeseen experiment circumstances.

Replication

Reproducibility analysis of magnetic susceptibility in patients (Fig. S7) performed by two raters (one was blinded). Bland-Altman and intra-class correlation coefficient show no significant bias and good correlation between raters.

Randomization

Swine (n=17) were randomized by time-to-reperfusion into four groups: reperfusion at 45 (n=3), 90 (n=5), 180 (n=5) minutes after infarct onset and permanent occlusion (n=4). Histology and spectrometry tissue samples were allocated into time-to-reperfusion groups then into viable myocardium and infarct groups. Infarct regions were depicted based on visual inspection and viable myocardium was collected in remote regions that were not impacted by ischemia.

Blinding

Cardiac structure, function and volumetric analysis were performed blinded for both animals and patients. Other MRI quantitative measurements and iron concentration measurements were unblinded.

Reporting for specific materials, systems and methods

We require information from authors about some types of materials, experimental systems and methods used in many studies. Here, indicate whether each material, system or method listed is relevant to your study. If you are not sure if a list item applies to your research, read the appropriate section before selecting a response.

Materials & experimental systems

n/a	Involved in the study
☒	☐ Antibodies
☒	☐ Eukaryotic cell lines
☒	☐ Palaeontology
☐	☒ Animals and other organisms
☐	☒ Human research participants
☐	☒ Clinical data

Methods

n/a	Involved in the study
☒	☐ ChIP-seq
☒	☐ Flow cytometry
☒	☐ MRI-based neuroimaging

Animals and other organisms

Policy information about studies involving animals; ARRIVE guidelines recommended for reporting animal research

Laboratory animals	Yorkshire swine (N=38; 30-35 kg at baseline, 100% male).
Wild animals	No wild animals were used in the study.
Field-collected samples	No field collected samples were used in the study.
Ethics oversight	Yorkshire swine were studied according to the protocols approved by the Institutional Animal Care and Use Committee of University of Pennsylvania (Philadelphia, PA).

Note that full information on the approval of the study protocol must also be provided in the manuscript.

Human research participants

Policy information about studies involving human research participants

Population characteristics	Patients (n=7, age=61±9 years, 1 female) enrolled in this study had ST-elevation acute myocardial infarctions and were treated by primary percutaneous coronary intervention (PCI). MRI studies were performed on a 1.5 T whole-body system (Aera; Siemens Healthcare, Erlangen, Germany) within 48 hours of PCI therapy.
Recruitment	Eligibility criteria included: (1) between the ages of 18 and 80, (2) must be able to read and understand English and sign the informed consent form, (3) elevated and delayed peak creatine kinase-MB and troponin I (cTnI) and troponin T (cTnT) in blood serum, (4) no contraindications to magnetic resonance imaging such as claustrophobia, (5) adequate renal function, as determined by glomerular filtration rate >30 ml/min, (6) no presence of cardiac pacemaker or implanted cardioverter defibrillator, (7) were not pregnant, (8) did not have a personal or family history of hypertrophic cardiomyopathy, (9) did not have a history of seizure disorder. The indications for PCI were based on electrocardiogram criteria showing 1 mm ST elevation in precordial leads or 0.5 mm ST elevation in limb leads in at least 2 contiguous leads. A minority of patients received thrombolytic therapy. The indications for PCI in this case were: (1) lack of resolution of ECG changes, as determined by >50% resolution of ST elevation from maximum elevation, (2) significant resolution of chest pain, as assessed by verbal confirmation with the patients, and (3) hemodynamic instability, as determined by non-invasive blood pressure measurement, heart rate changes on ECG and clear lungs.
Ethics oversight	The clinical trial was conducted in accordance with the Declaration of Helsinki and the Guidelines for Good Clinical Practice. The trial protocol was approved by the Institutional Review Board of the University of Pennsylvania (Philadelphia, PA, USA).

Note that full information on the approval of the study protocol must also be provided in the manuscript.

Clinical data

Policy information about clinical studies

All manuscripts should comply with the ICMJEguidelines for publication of clinical research and a completedCONSORT checklist must be included with all submissions.

Clinical trial registration	NCT03531151 MRI of Myocardial Infarction
Study protocol	Full trial protocol is available upon reasonable request to the authors and approved through the University of Pennsylvania. There may be restrictions on private health data. Protocol can be accessed through ClinicalTrials.gov
Data collection	Data was collected at the hospital of the University of Pennsylvania.
Outcomes	Primary: ejection fraction (<36 hours to >6 months) Secondary: relaxation times and extracellular volume fraction
